# Supplementary material for: The photoswitchable cannabinoid azo‐HU308 enables optical control of Ca2+ dynamics in INS‐1 β‐cells via off‐target effects on TRPC channels
Source: FEBS Open Bio. 2025 Nov 2;16(3):520–31. doi: 10.1002/2211-5463.70146 (PMC12955749; doi:10.1002/2211-5463.70146)
Supplement: Supplementary file 1 — Fig. S1. azo‐HU308 increases Ca2+ transient in INS‐1 cells with stimulated by UV‐A light. Fig. S2. Nonphotoswitchable HU308 addition causes a short Ca2+ transient in INS‐1 cells. Fig. S3. azo‐HU308's photoswitching effect on Ca2+ is not CB1‐ or GPR55‐mediated. Fig. S4. azo‐HU308's photoswitching effect is not TRPV1‐ or TRPC6‐mediated. Table S1. Statistical significance calculations for Fig. 1E. Table S2. Statistical significance calculations for Fig. S1C. Table S3. Statistical significance calculations for Fig. 2A. Table S4. Statistical significance calculations for Fig. 3A. Table S5. Statistical significance calculations for Fig. 4A. [file FEB4-16-520-s001.docx]

**The photoswitchable cannabinoid *azo*-HU308 enables optical control of Ca^2+^ dynamics in INS-1 β-cells via off-target effects on TRPC channels**

Alexander E.G. Viray^1^, James A. Frank^1,2^*

^1^ Department of Chemical Physiology and Biochemistry, Oregon Health & Science University, 3181 SW Sam Jackson Park Road, Portland, OR 97239-3098, USA

^2^ Vollum Institute, Oregon Health & Science University, 3181 SW Sam Jackson Park Road, Portland, OR 97239-3098, USA

*Correspondence: frankja@ohsu.edu

**SUPPORTING INFORMATION**

**SUPPORTING FIGURES**

**­**
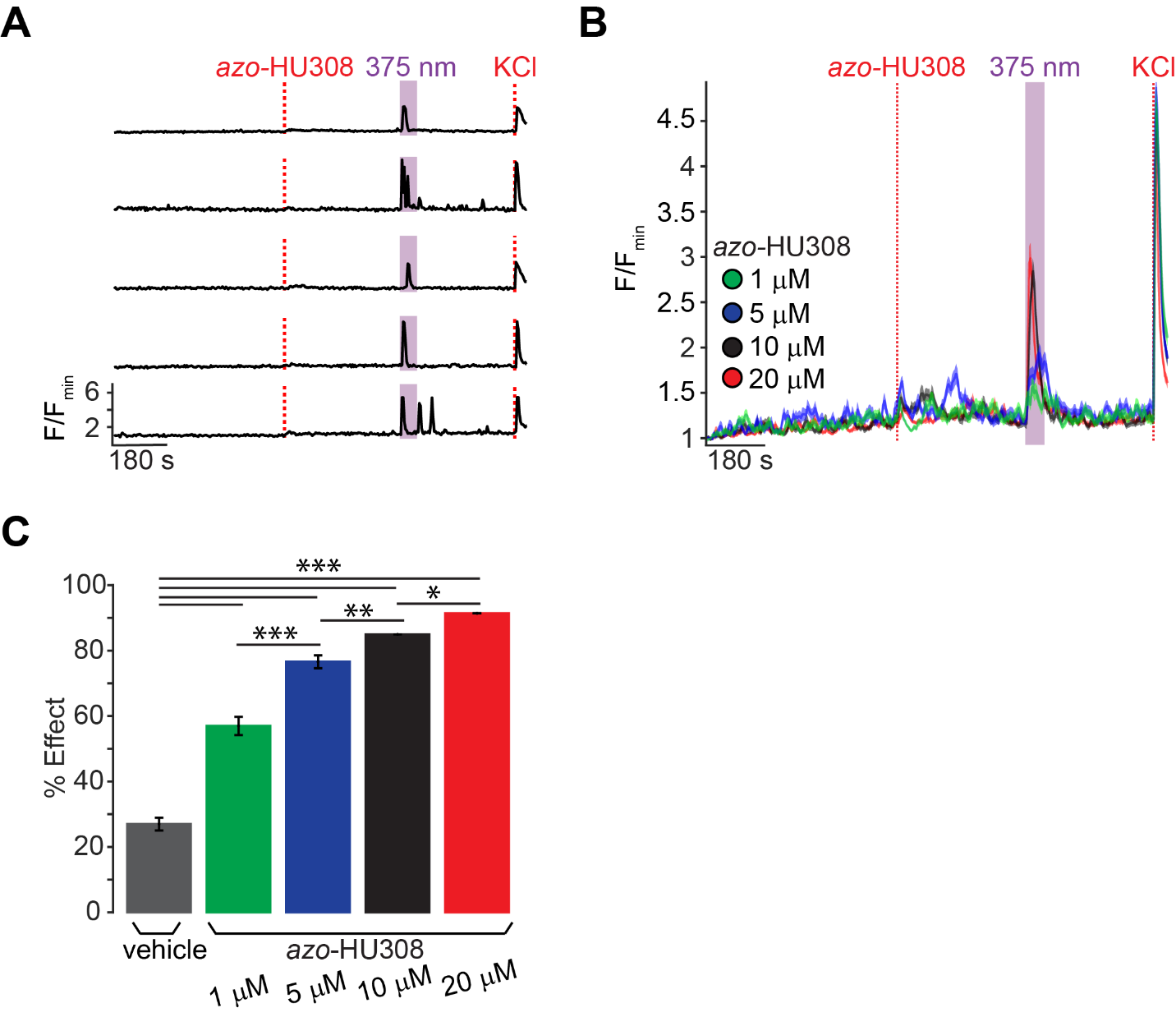


**FIGURE S1. *azo*-HU308 increases Ca^2+^ transient in INS-1 cells with stimulated by UV-A light.** INS-1 cells were transfected with RGECO and their Ca^2+^ levels monitored by confocal microscopy. (**A**) Representative single cell Ca^2+^ traces for INS-1 cells after the addition of *azo*-HU308 (20 μM), which caused an increase in Ca^2+^ after photoswitching to *cis* with 375 nm light. KCl (30 mM) was added at the end of each experiment to maximize intracellular Ca^2+^. (**B**) Averaged Ca^2+^ imaging traces showing dose-response for *azo*-HU308 at 1μM (N = 157, T = 3), 5 μM (N = 162 cells, T = 3), 10 μM (N = 149, T = 3), and 20 μM (N = 170, T = 4) followed by photoactivation with 375 nm light. (**C**) Summary bar graph comparing Ca2+ responses to the vehicle (N = 203, T = 4) and dose-response of *azo*-HU308 as shown in part B. Error bars = mean ± s.e.m. *P < 0.05, **P < 0.01, ***P < 0.001, ns = not significant = P > 0.05. Student’s T-Test.


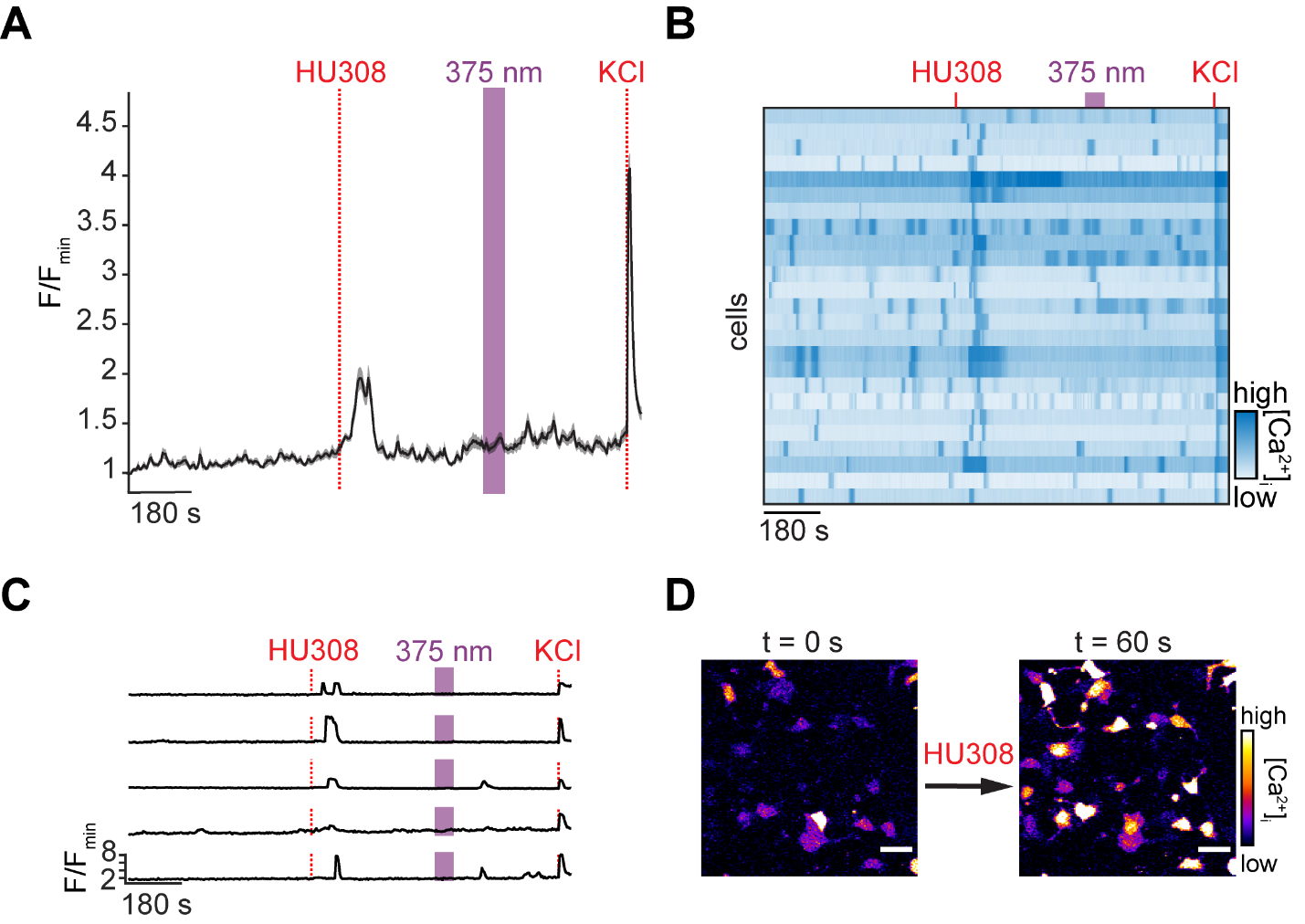


**FIGURE S2.** **Non-photoswitchable** **HU308 addition causes a short Ca^2+^ transient in INS-1 cells.** INS-1 cells were transfected with RGECO and their Ca^2+^ levels monitored by confocal microscopy. (**A**) Averaged (N = 150, T = 3), (**B**) heatmap of 25 cells, and (**C**) representative single cell Ca^2+^ traces for INS-1 cells after the addition of HU308 (20 μM). An increase in Ca^2+^ was observed upon addition, but no response was triggered upon 375 nm irradiation. For A, shaded error bars = mean ± s.e.m.. For B, Ca^2+^ traces from 25 representative cells were normalized to the KCl response. (**D**) Representative fluorescence micrographs showing increased Ca^2+^ in INS-1 cells after HU308 addition. Scale bar = 30 μm.


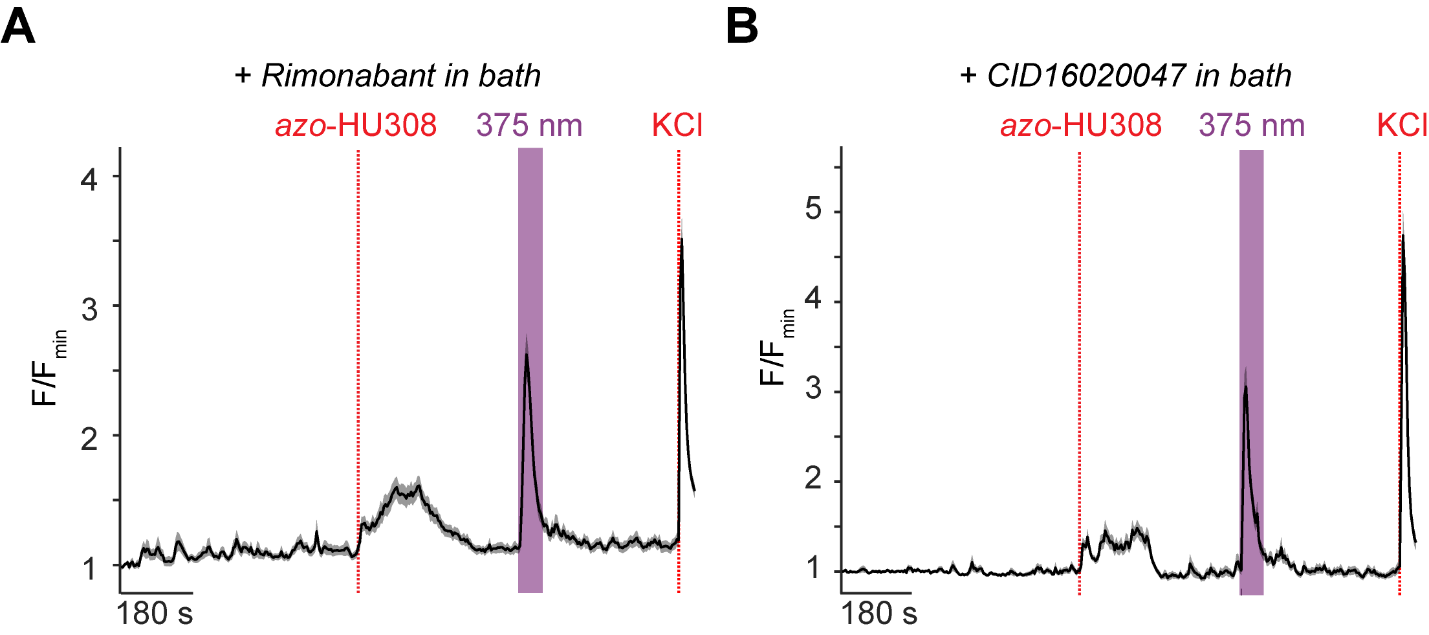


**FIGURE S3.** ***azo*-HU308’s photoswitching effect on Ca^2+^ is not CB1- or GPR55-mediated.** INS-1 cells were transfected with RGECO and their Ca^2+^ levels monitored by confocal microscopy. (**A,B**) Averaged Ca^2+^ imaging traces showing the effect of *azo*-HU308 (20 μM) addition and 375 nm photostimulation in the presence of (A) the CB1 antagonist rimonabant (2 μM, N = 103, T = 2) and (B) the GPR55 antagonist CID16020047 (20 μM, N = 104, T = 2). A Ca^2+^ increase was still observed on addition and stimulation. Shaded error bars = mean ± s.e.m.


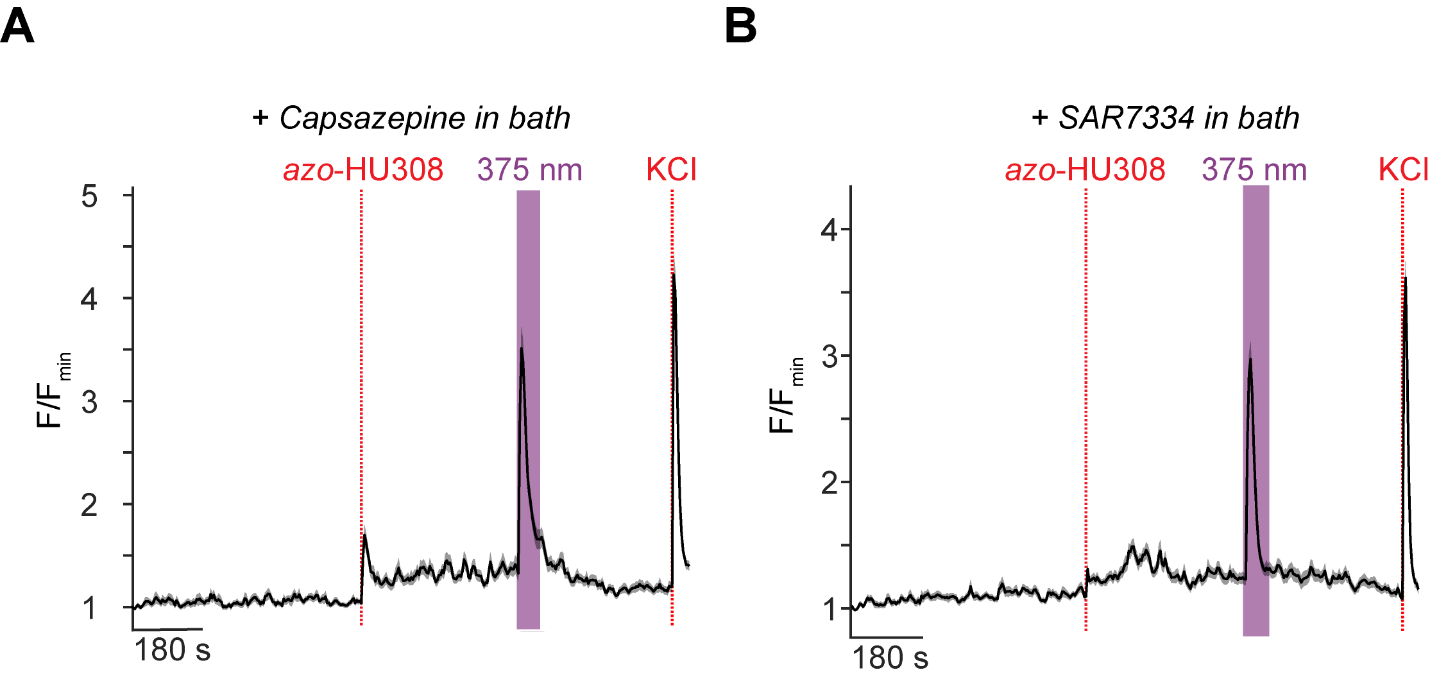
 **FIGURE S4. *azo*-HU308’s photoswitching effect is not TRPV1- or TRPC6-mediated.** INS-1 cells were transfected with RGECO and their Ca^2+^ levels monitored by confocal microscopy. (**A,B**) Average Ca^2+^ imaging traces showing that *azo*-HU308 addition and photostimulation with 375 nm light still had an effect on Ca^2+^ in the presence of (A) the TRPV1 blocker Capsazepine (5 μM, N = 119, T = 2) and (B) the TRPC6 blocker SAR7334 (20 μM, N = 164, T = 3). Shaded error bars = mean ± s.e.m.

**SUPPORTING TABLES**

**TABLE S1: Statistical significance calculations for Figure 1E**

| **conditions** | **p-value** | **significance** |
| --- | --- | --- |
| *trans-azo-*HU308 */ cis-azo*-HU308 | 5.32E-68 | *** |
| HU308 + 375 nm / *cis-azo-*HU308 | 1.55E-48 | *** |
| vehicle + 375 nm / *cis azo-*HU308 | 8.41E-74 | *** |
| vehicle + 375 nm / HU308 + 375 nm | 0.0238 | * |
| HU308 dark */* HU308 + 375 nm | 2.47E-07 | *** |
| HU308 dark */ cis-azo-*HU308 | 1.80E-11 | *** |
| vehicle dark */ cis-azo-*HU308 | 0.1169 | n.s. |
| vehicle dark */* HU308 + 375 nm | 5.91E-11 | *** |
| vehicle dark */* vehicle + 375 nm | 0.0139 | * |

***P<0.005 (dark blue, *P<0.05 (light blue), ns = P>0.05 (white)

**TABLE S2: Statistical significance calculations for Figure S1C**

| **conditions** | **p-value** | **significance** |
| --- | --- | --- |
| *-cis* 10μM *azo-*HU308 / *-cis* 20μM *azo*-HU308 | 0.0227 | n.s. |
| *-cis* vehicle / *-cis* 10μM *azo-*HU308 | 3.36E-61 | *** |
| *-cis* 1μM *azo-*HU308 / *-cis* 5μM *azo-*HU308 | 2.01E-08 | *** |
| *-cis* vehicle / *-cis* 1μM *azo-*HU308 | 8.02E-18 | *** |
| *-cis* vehicle / *-cis* 20μM *azo-*HU308 | 8.41E-74 | *** |
| *cis* 5μM *azo-*HU308 / *-cis* 10μM *azo-*HU308 | 0.0032 | *** |
| *-cis* vehicle / *-cis* 5μM *azo-*HU308 | 1.48E-50 | *** |

***P<0.01 (dark blue, *P<0.05 (light blue), ns = P>0.05 (white)

**TABLE S3 Statistical significance calculations for Figure 2A**

| **conditions** | **p-value** | **significance** |
| --- | --- | --- |
| *-cis azo*-HU308 + AM630 | 0.1564 | n.s. |
| *-cis azo*-HU308 + CID16020047 | 0.0378 | * |
| *-cis azo*-HU308 + YM254890 | 6.62E-25 | *** |
| *-cis azo*-HU308 + JWH133 | 0.8940 | n.s. |
| *-cis azo*-HU308 + NF449 | 0.0305 | * |
| *-cis azo*-HU308 + Pertussis Toxin | 3.71E-04 | *** |
| *-cis azo*-HU308 + Rimonabant | 0.3640 | n.s. |
| *-cis azo*-HU308 + SR144528 | 4.83E-04 | *** |
| *-cis azo*-HU308 + U73122 | 0.4006 | n.s. |
| *-cis azo*-HU308 + Xestospongin C | 0.1982 | n.s. |

***P<0.01 (dark blue, *P<0.05 (light blue), ns = P>0.05 (white)

**TABLE S4: Statistical significance calculations for Figure 3A**

| **conditions** | **p-value** | **significance** |
| --- | --- | --- |
| *-cis azo*-HU308 + 100μM 2-APB | 1.74E-75 | *** |
| *-cis azo*-HU308 + 1μM 2-APB | 0.4480 | n.s. |
| *-cis azo*-HU308 + 50μM 2-APB | 3.03E-31 | *** |
| *-cis azo*-HU308 + Ca^2+^ free | 1.06E-133 | *** |
| *-cis azo*-HU308 + Capsazepine | 0.3447 | n.s. |
| *-cis azo*-HU308 + ML204 | 1.80E-26 | *** |
| *-cis azo*-HU308 + SAR7334 | 0.1265 | n.s. |
| *-cis azo*-HU308 + SKF966365 | 0.0829 | n.s. |

***P<0.01 (dark blue, *P<0.05 (light blue), ns = P>0.05 (white)

**TABLE S5: Statistical significance calculations for Figure 4A**

| **conditions** | **p-value** | **significance** |
| --- | --- | --- |
| *-cis azo*-HU308 + Pyr10 | 1.26E-30 | *** |
| *-cis azo*-HU308 + siRNAscramble | 0.0248 | * |
| *-cis azo*-HU308 + siRNATRPC3 | 1.73E-20 | *** |
| *-cis azo*-HU308 + YM58483 | 1.58E-113 | *** |
| *-cis azo*-HU308 + Pyr10 /  *-cis azo*-HU308 + siRNATRPC3 | 1.54E-05 | *** |
| *-cis azo*-HU308 + siRNA low GC scramble */*  *-cis azo*-HU308 + siRNATRPC3 | 0.007 | *** |

***P<0.01 (dark blue, *P<0.05 (light blue), ns = P>0.05 (white)
